# Genome-Wide Identification, Characterization, and Expression Analysis of the HD-Zip Gene Family in Quinoa (*Chenopodium quinoa*)

**DOI:** 10.3390/biology15212012

**Published:** 2026-11-05

**Authors:** Pibiao Shi, Runzhi Jiang, Deling Wang, Zhenmeng Ji, Bin Li, Minfeng Gu

**Affiliations:** Xinyang Agricultural Experiment Station of Yancheng City, Jiangsu Academy of Agricultural Sciences, Yancheng 224049, China

**Keywords:** HD-Zip, gene family, bioinformatics, gene expression, abiotic stress, *Chenopodium quinoa*

## Abstract

The homeodomain-leucine zipper (HD-Zip) proteins are important transcription factors in the regulation of plant growth and development and abiotic stress responses. However, our understanding of the HD-Zip family is still limited in quinoa. In this study, 41 *CqHD-Zip* genes were identified in the quinoa genome and then phylogenetically classified into four subfamilies. Their characterizations, chromosomal locations, gene structures, conserved motifs, and phylogenetic relationships were comprehensively analyzed through genome-wide bioinformatics methods. Our RNA-Seq data showed that a subset of the 41 *HD-Zip* genes exhibited expression changes under salt stress, among which *CqHDZ5*, *CqHDZ12*, *CqHDZ13*, *CqHDZ18*, *CqHDZ19,* and *CqHDZ21* responded positively to salt stress, and the results were confirmed by qRT-PCR analysis. Tissue expression revealed that *CqHDZ5*, *CqHDZ13*, *CqHDZ18,* and *CqHDZ21* were significantly expressed in developing seeds, and *CqHDZ12* and *CqHDZ19* were highly expressed in roots. Additionally, the transcript levels of these six genes were significantly up-regulated in response to osmotic and low-temperature stress, indicating that they may participate in stress resistance against salt, osmosis, and low temperature. It is shown that HD-Zip is a multifunctional gene family, and the results will provide valuable information for further analysis of the potential role of *HD-Zip* genes in abiotic stress responses and genetic improvement of quinoa.

## Simple Summary

Quinoa is a nutrient-dense crop that tolerates saline and other adverse growing conditions. Nevertheless, little is understood about the key internal genetic regulators enabling it to survive environmental stress. This work aimed to identify related genes in quinoa and investigate their biological roles. We uncovered 41 such genes, six of which showed strongly increased activity upon exposure to salt, drought-simulating, and cold conditions. Several of these genes function predominantly within developing seeds, whereas others are most active in plant roots. These results deepen our knowledge of quinoa’s stress-adaptation mechanisms and assist future breeding efforts to develop more stress-resilient quinoa varieties for agricultural production.

## 1. Introduction

Plant growth and development are often affected by a variety of adverse environmental factors, such as drought, soil salinity, and extreme temperatures. To adapt to these unfavorable environmental conditions, plants have evolved a series of regulatory mechanisms to respond to different stress signals [1,2]. These processes often lead to morphological and physiological changes in plants, which are controlled by complex gene regulatory networks [3]. As central regulators of gene expression, transcription factors play important roles in plant growth, development, and abiotic stress response [4]. Transcription factors are a group of DNA-binding proteins that can specifically bind to *cis*-acting elements of eukaryotic gene promoters to activate or inhibit the expression of downstream target genes. To date, more than 60 transcription factor families have been identified in plants [5]. The homologous domain leucine zipper (HD-Zip) gene family, one of the plant-specific transcription factor families, regulates various biological processes including plant growth and development, cell division, and responses to abiotic stresses [6,7]. HD-Zip proteins comprise a C-terminal homeodomain (HD) and an adjacent leucine zipper (LZ) domain. The HD can specifically bind to DNA, whereas the LZ domain mediates the formation of protein dimers [8]. Based on sequence conservation, gene structure, and cis-elements, the HD-Zip gene family can be divided into four subfamilies: HD-Zip I, II, III, and IV [9]. HD-Zip I and HD-Zip II members possess both HD and LZ domains, which could specifically bind to the dual symmetric DNA sequence of CAATNATTG [10]. Additionally, HD-Zip II proteins contain a conserved CPSCE (Cys-Pro-Ser-Cys-Glu) motif and N-terminal consensus sequences. Except for HD and LZ domains, the HD-Zip III and HD-Zip IV subfamilies possess a steroidogenic acute regulatory protein-related lipid transfer (START) domain and a START-associated domain (SAD) [11,12]. Different from HD-Zip IV, the HD-Zip III proteins share a common methionine-glutamic-lysine-histidine-leucine-alanine (MEKHLA) domain related to various chemical and physical stimuli [13]. Additionally, the genes of the same subfamily often exhibit functional conservation due to structural similarity.

Since the first HD-containing gene Knotted1 was isolated in maize [14], numerous HD-Zip transcription factors have been identified by genome-wide analysis in different species, such as pepper [15], grape [16], foxtail millet [17], watermelon [18], wheat [19], Chinese chestnut [20], and peach [21]. Moreover, the crucial roles of this family of genes in multiple biological processes of plants have also been reported by more researchers. Analysis of mutant and transgenic plants revealed that AtHB12, one HD-Zip I member, accelerates seedling root elongation and leaf development [22]. Tomato HD-Zip transcription factor SlHB15A could regulate pedicel abscission by modulating jasmonoyl-isoleucine biosynthesis [23], and the HD-Zip IV proteins could modify floral structures for self-pollination [24]. Overexpression of AtHB13 and HaHB1 can remarkably enhance the cold tolerance of transgenic plants via the induction of proteins able to stabilize cell membranes and inhibit ice growth [25]. Ectopic expression of an HD-Zip I family gene from physic nut, JcHDZ21, decreased the salt resistance of transgenic Arabidopsis, suggesting that JcHDZ21 was a negative regulator in regulating plant responses to salt stress [26]. In cucumber, the silencing of CsGL2-LIKE, an HD-Zip IV transcription factor, delayed male flowering by suppressing the expression of the florigen gene FT and reduced pollen viability and seed vigor [27]. Two HD-Zip proteins, GLABRA2 (GL2) and HOMEODOMAIN GLABROUS11 (HDG11), play redundant functions in Arabidopsis trichomes, and GL2 activates a positive feedback loop via MYB23 [28]. Overexpression of TaHDZipI-5 improved transgenic wheat freezing and drought tolerance [29]. ZmHDZ10 overexpression in both rice and Arabidopsis positively regulated drought and salt tolerance through altering the expression of stress/ABA-responsive genes, including P5CS1, RD22, RD29B, and ABI1 [30]. Cotton HD-Zip I transcription factor GhHB4-like-GhNAC007 regulated plant response to salt stress, potentially by modulating GhMYB96 and ABA signaling-related genes [31]. These studies suggested that the HD-Zip gene family may play a vital role in plant resistance to abiotic stress.

Quinoa (*Chenopodium quinoa* Willd.) is a dicotyledonous crop with abundant nutritional elements, originating from the Andean area of South America, belonging to the Chenopodiaceae family along with sugar beet (*Beta vulgaris* L.) and spinach (*Spinacia oleracea* L.) [32,33]. Many consumers favor quinoa because of its nutritional values and health-promoting properties such as antioxidant, anti-obesity, anticancer, antimicrobial, antidiabetic, and cardio-beneficial. Quinoa has become more and more popular among researchers due to its extraordinary adaptability to extreme climate and soil conditions. However, little is known about the HD-Zip gene family in quinoa. In this study, the HD-Zip members in quinoa were identified, and we analyzed their protein physicochemical properties, chromosome distributions, phylogenetic relationships, gene structures, and motif locations. Additionally, we performed transcriptome sequencing of quinoa roots under salt stress, and six genes of this family were found to be up-regulated, which were also confirmed by qRT-PCR analysis. Then, the expression patterns of these genes under osmotic and low-temperature stress and in different tissues were examined. These findings provide insights for a more systematic understanding of the characterization of the *CqHD-Zip* genes and contribute to the exploration of the functions and molecular breeding of abiotic stress tolerance in quinoa.

## 2. Materials and Methods

### 2.1. Identification and Sequence Analysis of HD-Zip Gene Family in Quinoa

Using the protein sequences of the Arabidopsis HD-Zip gene family retrieved from the TAIR database (http://www.arabidopsis.org, accessed on 8 July 2026) as query sequences, the quinoa genome database was searched using local BLASTP with an E-value < 10^−5^ (https://phytozome-next.jgi.doe.gov/info/Cquinoa_v1_0, accessed on 9 July 2026). Then the SMART web tool (http://smart.emblheidelberg.de, accessed on 12 July 2026) and the NCBI CDD search (http://www.ncbi.nlm.nih.gov/Structure/cdd/wrpsb.cgi, accessed on 13 July 2026) were used to confirm the presence of the HD (PF00046)/LZ (PF02183)/START (PF01852)/MEKHLA (PF08670) domain in the resulting 41 non-redundant CqHD-Zip protein sequences with the E value < 10^−3^. The ExPASy ProtParam tool (http://web.expasy.org/protparam/, accessed on 17 July 2026) was used to analyze the protein length, molecular weight, and isoelectric point of each CqHD-Zip protein. The open reading frames of the *CqHDZs* were determined by the Open Reading Frame Finder tool (https://www.ncbi.nlm.nih.gov/orffinder/, accessed on 22 July 2026). Subcellular localization prediction was performed using the PSORT WWW Server (https://psort.hgc.jp/, accessed on 23 July 2026). The chromosomal distribution of *CqHDZs* was visualized by TBtools-II (V2.096) software (https://github.com/CJ-Chen/TBtools, accessed on 26 July 2026).

### 2.2. Multiple Sequence Alignment and Phylogenetic Tree Construction

The full-length sequences of HD-Zip proteins from quinoa (CqHDZs) and Arabidopsis (AtHDZs) were aligned with ClustalW with default parameters. A neighbor-joining (NJ) phylogenetic tree was constructed using MEGA 5.0 based on the alignment. Additionally, the statistical parameters used to build the NJ tree were as follows: Poisson model, complete deletion, and 1000 bootstrap replicates.

### 2.3. Gene Structure and Conserved Motif Analysis

The exon-intron structures of the CqHDZs were determined based on their ORF sequences and corresponding genomic sequences, and visualized using the online program Gene Structure Display Server (GSDS 2.0, http://gsds.gao-lab.org/, accessed on 27 July 2026). The Multiple Em for Motif Elicitation (MEME) online program (http://meme-suite.org/, accessed on 3 August 2026) was employed to analyze the conserved motifs of the CqHD-Zip proteins. The parameters for performing this analysis were as follows: The number of repetitions is any, the maximum number of motifs is 20, and the optimum motif width is between 6 and 50 amino acids, E-value < 10^−2^.

### 2.4. Expression Profiling of CqHD-Zip Genes

RNA-Seq raw data of roots at different periods of salt treatment in a hydroponic experiment were obtained from our previous research [34]. Gene expression levels were determined by the fragments per kilobase of transcript per million mapped reads (FPKM). The expression heatmap of 41 CqHDZs was visualized using HemI(version 1.0) software [35], and the ratios were log2-transformed.

### 2.5. Plant Materials and Abiotic Stress Treatments

In this study, the quinoa variety Suli No.1 from Jiangsu Academy of Agricultural Sciences was used as the experimental material. For tissue-specific expression analysis, roots, stems, and leaves were harvested from 28-day-old seedlings grown in the greenhouse. Growth continued to the reproductive stage until flowers and developing seeds were collected. The seeds were sampled at 10, 20, and 30 days after pollination (DAP). The seedlings grown hydroponically to the sixth true leaf stage were then subjected to abiotic stress treatments to determine the expression profiles of CqHDZs under different stress conditions. Stress treatments included salt treatment (300 mM NaCl), osmotic treatment (20% PEG 6000), and low temperature treatment (4 °C). The root samples were harvested at 0, 3, 6, 12, and 24 h after stress treatments, immediately frozen in liquid nitrogen, and stored at −80 °C for RNA isolation and cDNA synthesis. Three independent biological replicates were set for each sample.

### 2.6. RNA Isolation and qRT-PCR Analysis

Total RNA was extracted using RNeasy Plant Mini Kit (Qiagen, Hilden, Germany) and purified using RNase-Free DNase Set (Qiagen, Hilden, Germany) as per manufacturer’s instructions. cDNA was synthesized using SuperScript^TM^III First-Strand Synthesis SuperMix (Thermo Fisher Scientific, Waltham, MA, USA) with gDNA Eraser (Takara, Kusatsu, Shiga, Japan) following the manufacturer’s protocol. Real-time quantitative polymerase chain reaction (qRT-PCR) was performed using Power SYBR^®^ Green PCR Master Mix (Applied Biosystems, Foster City, CA, USA). The UBA2B was chosen as a reference gene. qRT-PCR reaction cycling conditions were set as follows: pre-denaturation at 95 °C for 1 min, then 40 cycles of 95 °C for 15 s, 60 °C for 25 s. The relative expression levels were calculated by the 2^−△△CT^ method [36]. The expression profiles were drawn using Origin 8 software.

## 3. Results

### 3.1. Identification of HD-Zip Genes in Quinoa

Using the HD-Zip gene family protein sequences of Arabidopsis as a reference, a BLASTP search was carried out in the quinoa genome database. Candidate proteins harboring the HD domain were retained for further phylogenetic classification. Proteins were assigned to HD-Zip subfamilies based on their phylogenetic position and conserved domain architecture. For canonical HD-Zip proteins, the combination of HD and LZ is expected; however, the LZ motif can be degenerate or truncated in some HD-Zip members and may not be detected by SMART or NCBI CDD. Sequences lacking complete diagnostic domain profiles were annotated as divergent HD homologs in Table S1. A total of 41 quinoa *HD-Zip* genes were obtained, and they were named *CqHDZ1* to *CqHDZ41* on the basis of their location on the chromosomes. The general characteristics of all the *CqHDZs*, including gene ID, chromosome location, number of exons, open reading frame (ORF) length, number of amino acids (AAs), molecular weight (MW), isoelectric point (pI), and prediction of subcellular localization, are listed in Table 1. The ORF length of the *CqHDZs* ranged from 549 bp (*CqHDZ33*) to 2547 bp (*CqHDZ37*), and the number of AAs was 182 to 848, respectively, with an average of 452. Protein MW varied from 20.91 kDa (CqHDZ33) to 92.30 kDa (CqHDZ37), and the pI ranged from 4.62 (CqHDZ32) to 9.37 (CqHDZ15). Subcellular localization results indicated that 33 CqHD-Zip proteins were located in the nucleus, 5 CqHD-Zip proteins (CqHDZ2, CqHDZ7, CqHDZ29, CqHDZ38, and CqHDZ40) were found in the cytoplasm, and 3 CqHD-Zip proteins (CqHDZ3, CqHDZ31, and CqHDZ37) were located in the chloroplast. This is only an in silico prediction and requires further experimental validation.

### 3.2. Phylogenetic Analysis and Group Classification of the CqHD-Zip Genes

**Figure 1:**
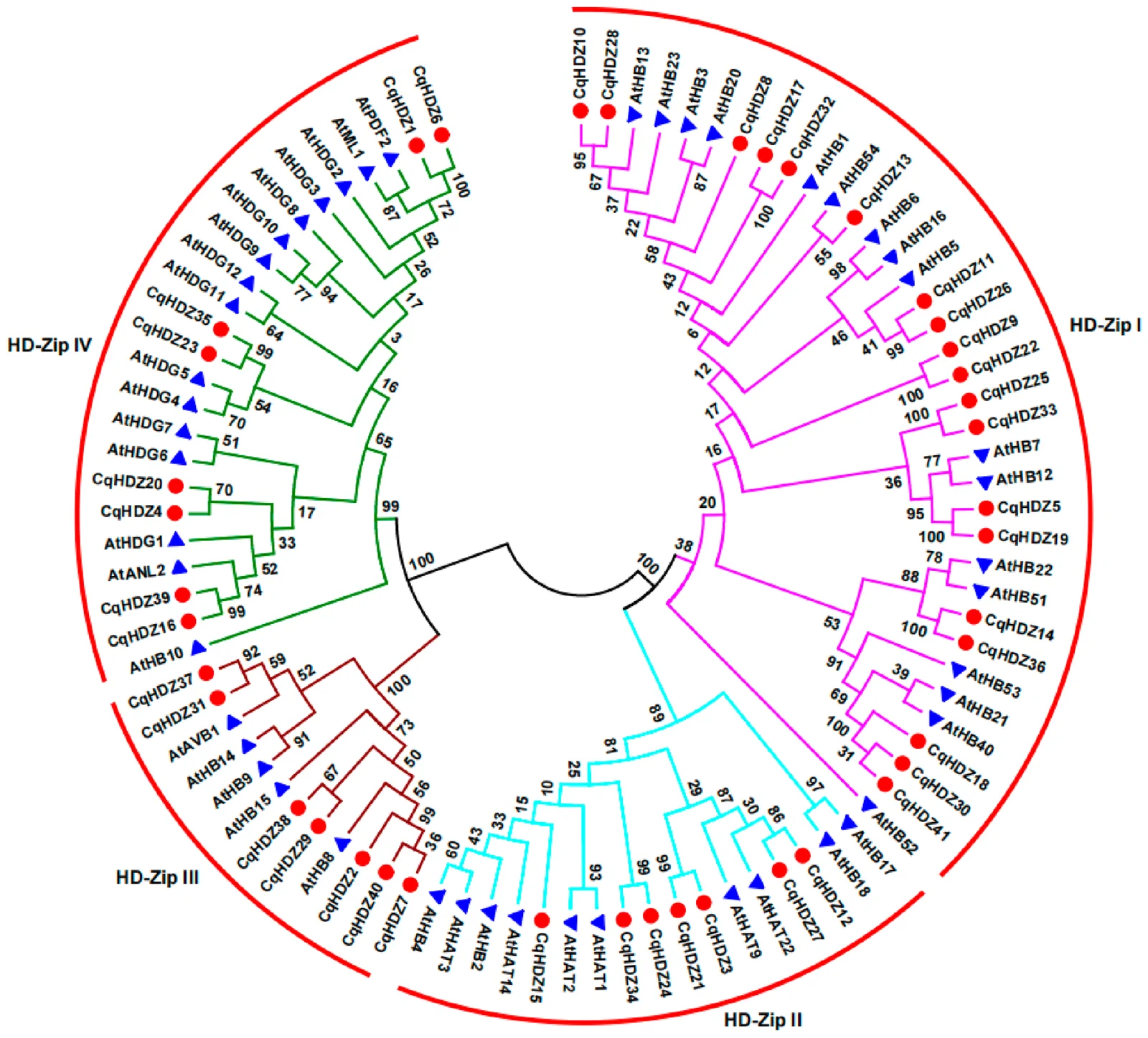
Phylogenetic analyses of HD-Zip proteins from quinoa and Arabidopsis. Neighbor-joining (NJ) method was adopted, and the bootstrap value was set to be equal to 1000. The tree shows the division of HD-Zip proteins into four subfamilies. Arabidopsis HD-Zip proteins are shown by a blue triangle, and quinoa HD-Zip proteins are shown by a red circle.

To assess the evolutionary relationship of the *CqHD-Zip* gene family, multiple sequence alignment of 41 CqHD-Zip proteins from quinoa and 48 AtHD-Zip proteins from Arabidopsis (Table S2) was performed; afterwards, a neighbor-joining (NJ) tree was constructed, and the phylogenetic relationship was analyzed. As shown in Figure 1, all 89 HD-Zip proteins were further divided into four subfamilies and were named I to IV, with 36 members in HD-Zip I, 17 in HD-Zip II, 12 in HD-Zip III, and 24 in HD-Zip IV. The 41 CqHDZs were distributed across all four subfamilies: HD-Zip II and HD-Zip III both had 7 members, HD-Zip IV had 8 members, while HD-Zip I had 19 members, which was the largest group. Generally, proteins in the same subfamily would have similar functions; hence, the clustering results of the phylogenetic tree could contribute to predicting the functions of CqHD-Zip proteins. For instance, CqHDZ13 and AtHB54 are classified into the same branch, suggesting that CqHDZ13 might have a similar biological function to AtHB54. In the HD-Zip I subfamily, CqHDZ5, CqHDZ19, CqHDZ25, CqHDZ33, AtHB7, and AtHB12 are in the same branch, exhibiting higher homology. Similarly, CqHDZ3, CqHDZ12, CqHDZ21, CqHDZ27, AtHAT9, and AtHAT22 have higher homology in HD-Zip II. CqHDZ7, CqHDZ29, CqHDZ38, CqHDZ40, AtHB8, and AtHB15 have higher homology in HD-Zip III. CqHDZ23 and CqHDZ35 were clustered with AtHDG4 and AtHDG5 in HD-Zip IV.

### 3.3. Chromosomal Distribution of the CqHD-Zip Genes

**Figure 2:**
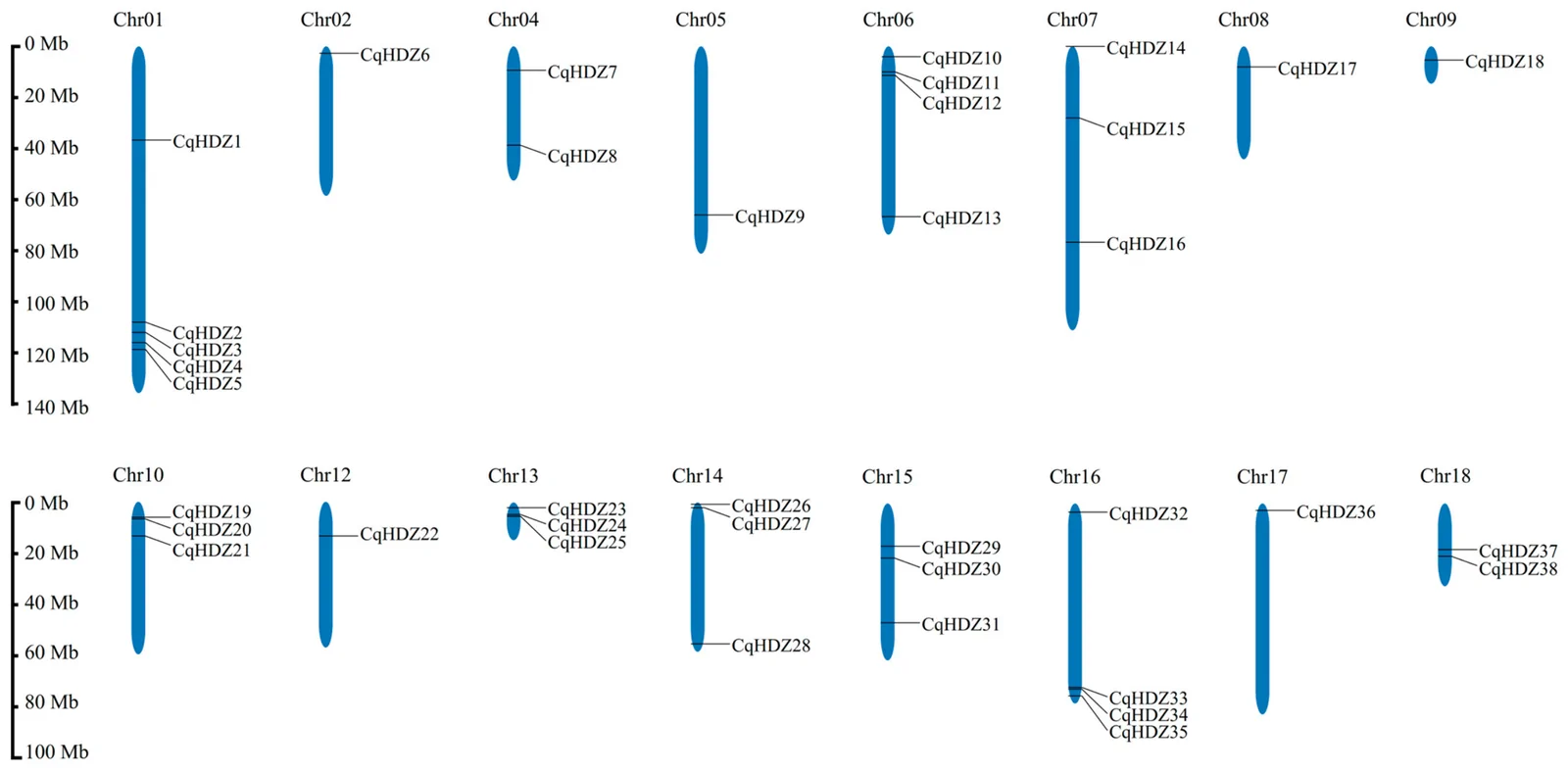
The chromosomal location of the *CqHD-Zip* genes.

The chromosome mapping results demonstrated that the *CqHD-Zip* genes were unevenly distributed on 16 of the 18 chromosomes (Chr) of quinoa, and most of them were located close to the distal positions of the chromosomes (Figure 2). *CqHD-Zip* genes were not found on Chr 3 and Chr11. *CqHDZ39*, *CqHDZ40*, and *CqHDZ41* were not accurately located on chromosomes and were named Chr 0 (Table 1). Additionally, it was also found that Chr1 contained five *CqHDZs*, which was the largest number of genes among these 16 chromosomes, followed by Chr6 and Chr16, which contained four. Six of the chromosomes (Chr2, Chr5, Chr8, Chr9, Chr12, and Chr17) carried only a single gene each, while two genes were located on Chr4 and Chr18, and three on Chr7, Chr10, Chr13, Chr14, and Chr15. Interestingly, some genes were densely distributed on the same chromosome, which might be related to gene replication events.

### 3.4. Gene Structure and Conserved Motif Analyses of CqHDZs

**Figure 3:**
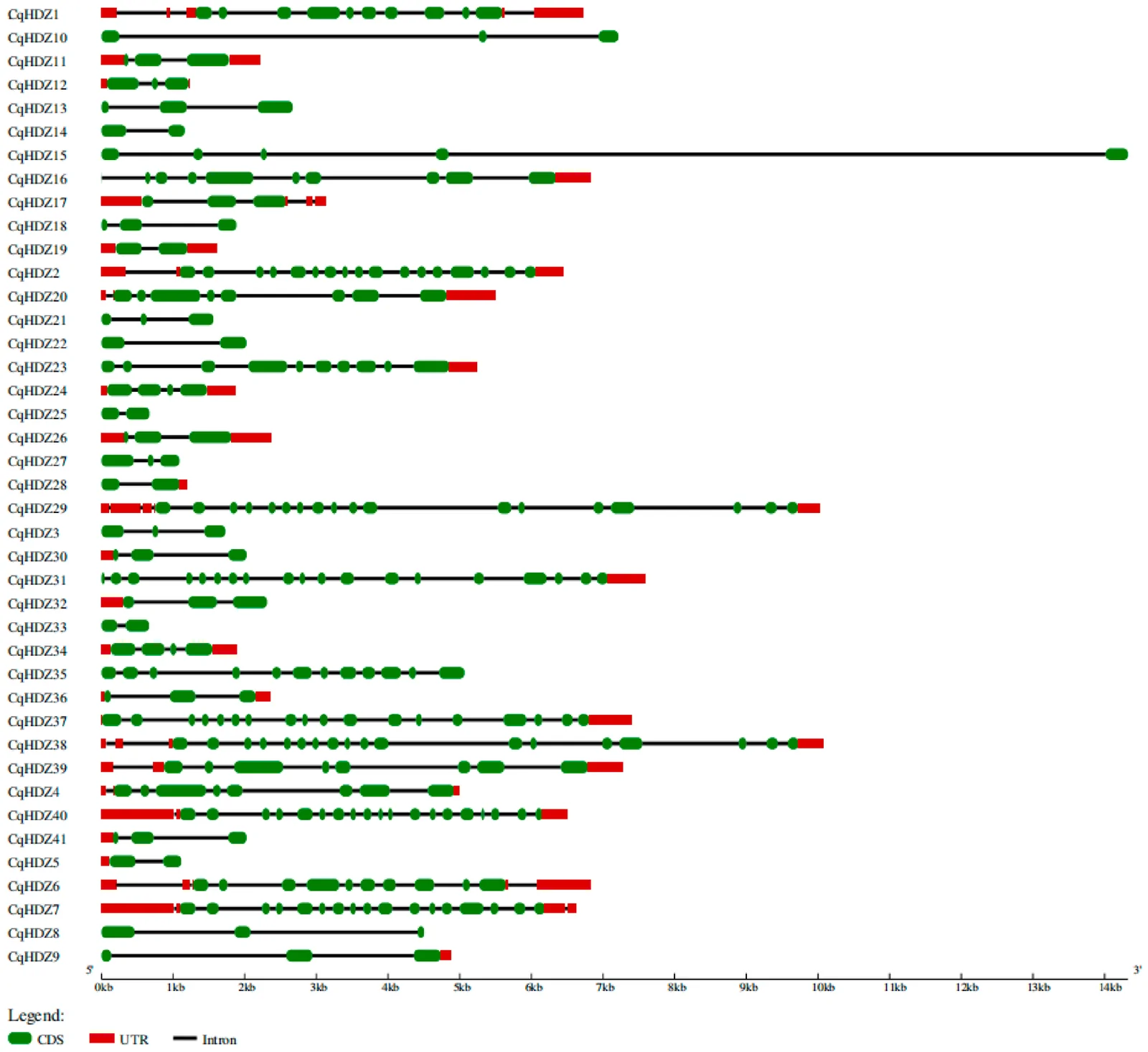
Gene structure of the *CqHD-Zip* genes. Green squares represent the coding sequence (CDS), red squares represent the untranslated region (UTR), and the black lines represent the intron.

To gain insights into the structure of the *CqHDZs*, we analyzed their intron and exon composition by comparing their genome sequences with corresponding cDNA sequences. It was found that the number of UTRs, introns, and exons varied greatly among different *CqHDZs* (Figure 3). The number of UTRs varies from 0 to 5, the number of introns is from 1 to 18, and the number of exons is from 2 to 19. The gene structure of the *CqHDZs* in HD-Zip I and HD-Zip II subfamilies is relatively simple, with the number of introns ranging from 1 to 2 and 2 to 4, respectively. The HD-Zip III subfamily possesses the most complicated gene structure, with 16 to 18 introns, followed by the HD-Zip IV subfamily with 7 to 11 introns. This could be due to the evolution of more diverse functional roles. However, genes in the same branch of the phylogenetic tree have structural similarities, such as *CqHDZ11* and *CqHDZ26* in the HD-Zip I subfamily containing two introns and three exons, and *CqHDZ24* and *CqHDZ34* in the HD-Zip II subfamily containing three introns and four exons, which further confirms the results of quinoa HD-Zip classification. Notably, CqHDZ30 and CqHDZ41 possess identical CDS sequences and are likely assembly redundancies derived from the unplaced scaffold Chr00.

**Figure 4:**
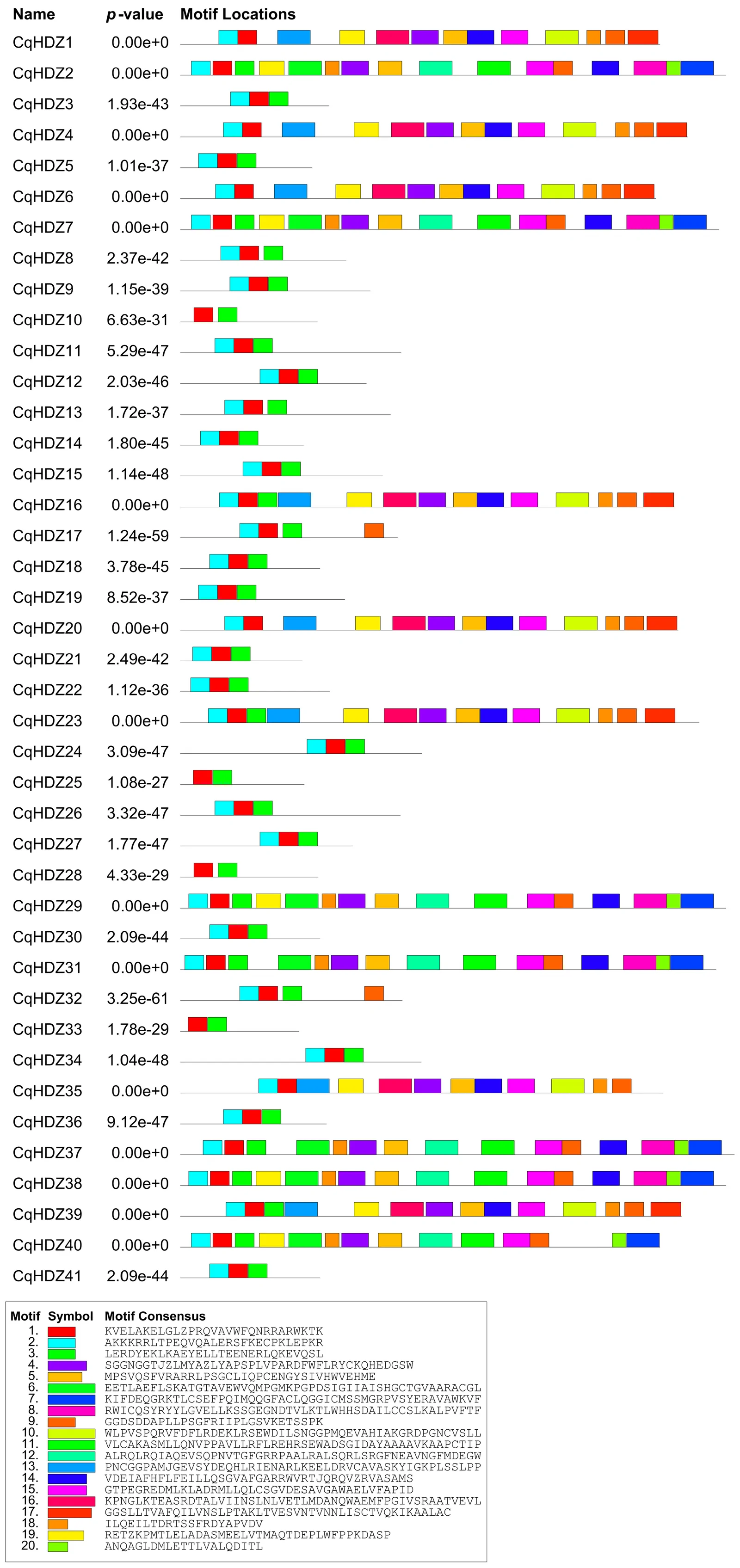
The conserved motifs of the CqHD-Zip proteins. Different colors indicate different motifs, and their consensus sequences are also listed.

In order to further study the conservation and differences in the protein sequences of CqHDZs, their conserved motifs were analyzed using MEME Suite Version 5.5.5 software. Twenty conserved motifs were finally determined, and their lengths varied from 21 to 50 amino acid residues (Figure 4). The results showed that the CqHD-Zip gene family members contained different numbers and types of motifs, but each contained motif 1 located in the HD domain, indicating the conservation of this family. The complexity of motif composition in different subfamilies is similar to the complexity of their gene structure, with HD-Zip III having the largest number of motifs (14 to 16), followed by HD-Zip IV (12 to 14). All the members in CqHD-Zip II have three motifs (motif 1, motif2, and motif3) located in the core domains HD and LZ. The differences in motif compositions and distributions among different genes predict their different biological functions. The findings that members from the same subfamily have similar structural compositions and cluster together strongly support the reliability of evolutionary tree classification.

### 3.5. Expression Profiles of CqHD-Zip Gene Family Under Salt Stress

**Figure 5:**
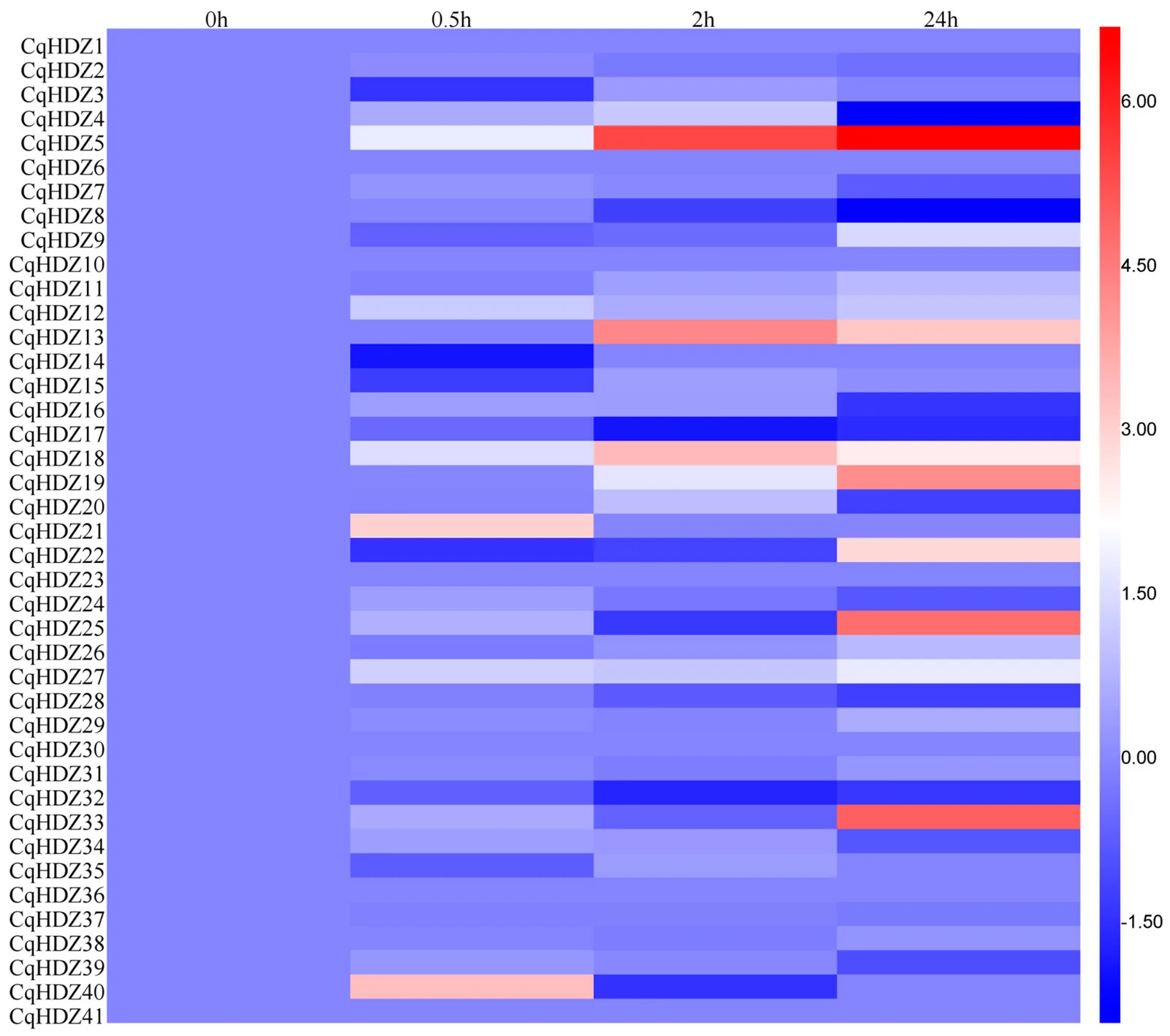
The expression heatmap of 41 *CqHD-Zip* genes at different time points under NaCl treatment as determined by RNA-Seq.

**Figure 6:**
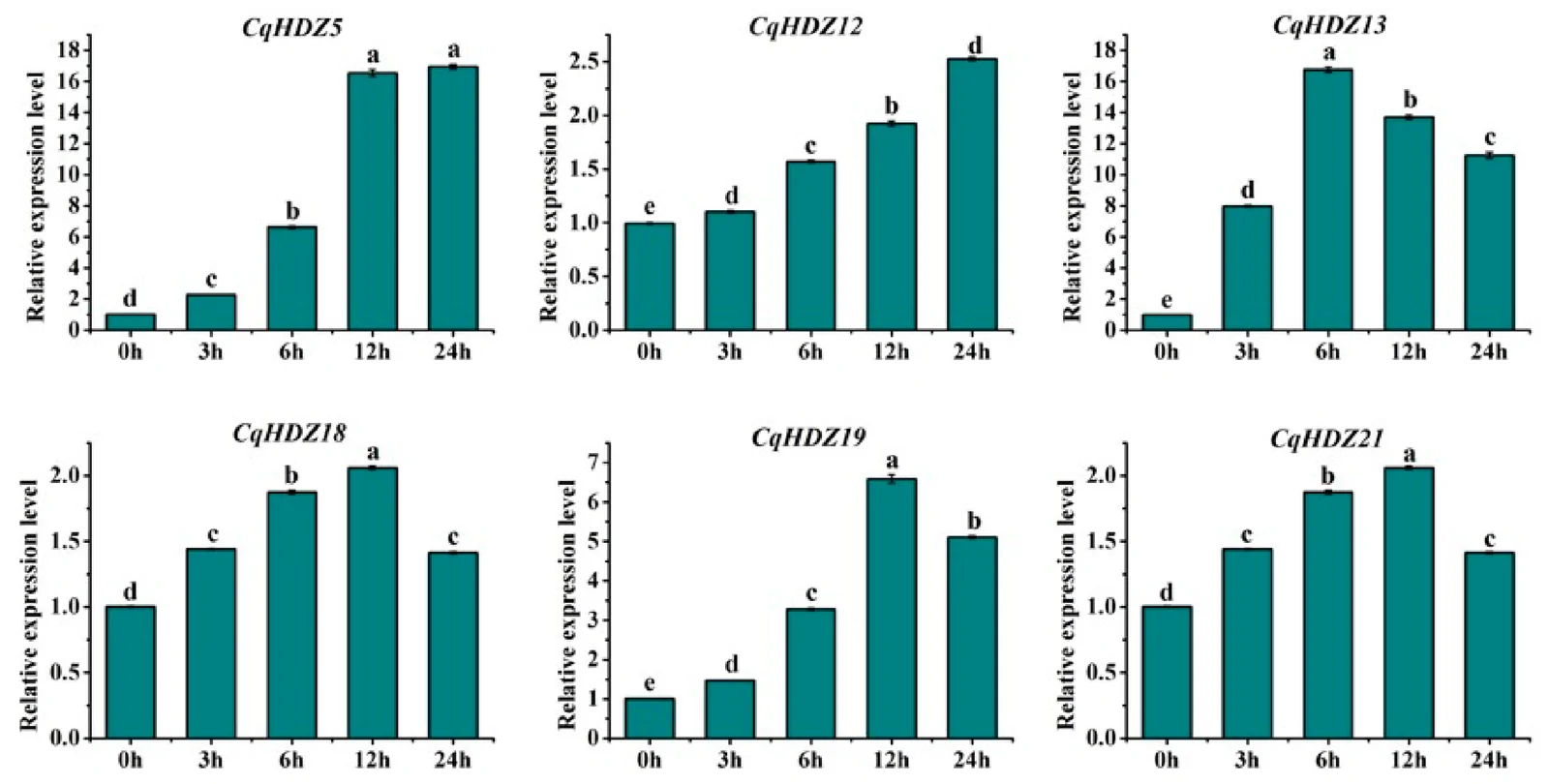
Expression analysis of six *CqHD-Zip* genes by qRT-PCR. The X-axis represents different time points of salt stress treatment; the Y-axis represents the relative expression levels. The data were normalized to the *UBA2B* gene, and the vertical bars indicate the mean values of three biological replicates ± standard deviation. Columns with different letters represent significant differences at the *p* < 0.05 level (Duncan’s test).

Combined with the transcriptome data from our previous research [34], the expression levels of *CqHD-Zip* genes treated with 300 mM NaCl at different time points were plotted into the heatmap as shown in Figure 5. There were certain differences in the expression patterns of CqHD-Zip family genes under salt stress. Among them, the expression levels of 7 genes, including *CqHDZ1*, *CqHDZ6*, *CqHDZ10*, *CqHDZ23*, *CqHDZ30*, *CqHDZ36,* and *CqHDZ41*, did not change, indicating that they may not participate in the response to salt stress, and the expression levels of the other 34 genes changed in varying degrees at different salt treatment time points. The expression of *CqHDZ9* and *CqHDZ22* was down-regulated at 0.5 h and 2 h, and up-regulated at 24 h, while the expression pattern of *CqHDZ4* was opposite. With the extension of salt treatment time, the expression levels of *CqHDZ25* and *CqHDZ33* increased first, then decreased, and then increased. In particular, *CqHDZ5*, *CqHDZ12*, *CqHDZ13*, *CqHDZ18*, *CqHDZ19* and *CqHDZ21* were induced by salt stress, while the expression levels of *CqHDZ8*, *CqHDZ17*, *CqHDZ28* and *CqHDZ32* were inhibited by salt treatment. To further validate the reliability of the RNA-Seq results, six salt-induced *CqHDZs* identified above were selected for qRT-PCR in the current study, which illustrated the consistency (Figure 6). In addition, the specific primers for qRT-PCR were listed in Table S3.

### 3.6. Tissue-Specific Expression Pattern of CqHD-Zip Genes

**Figure 7:**
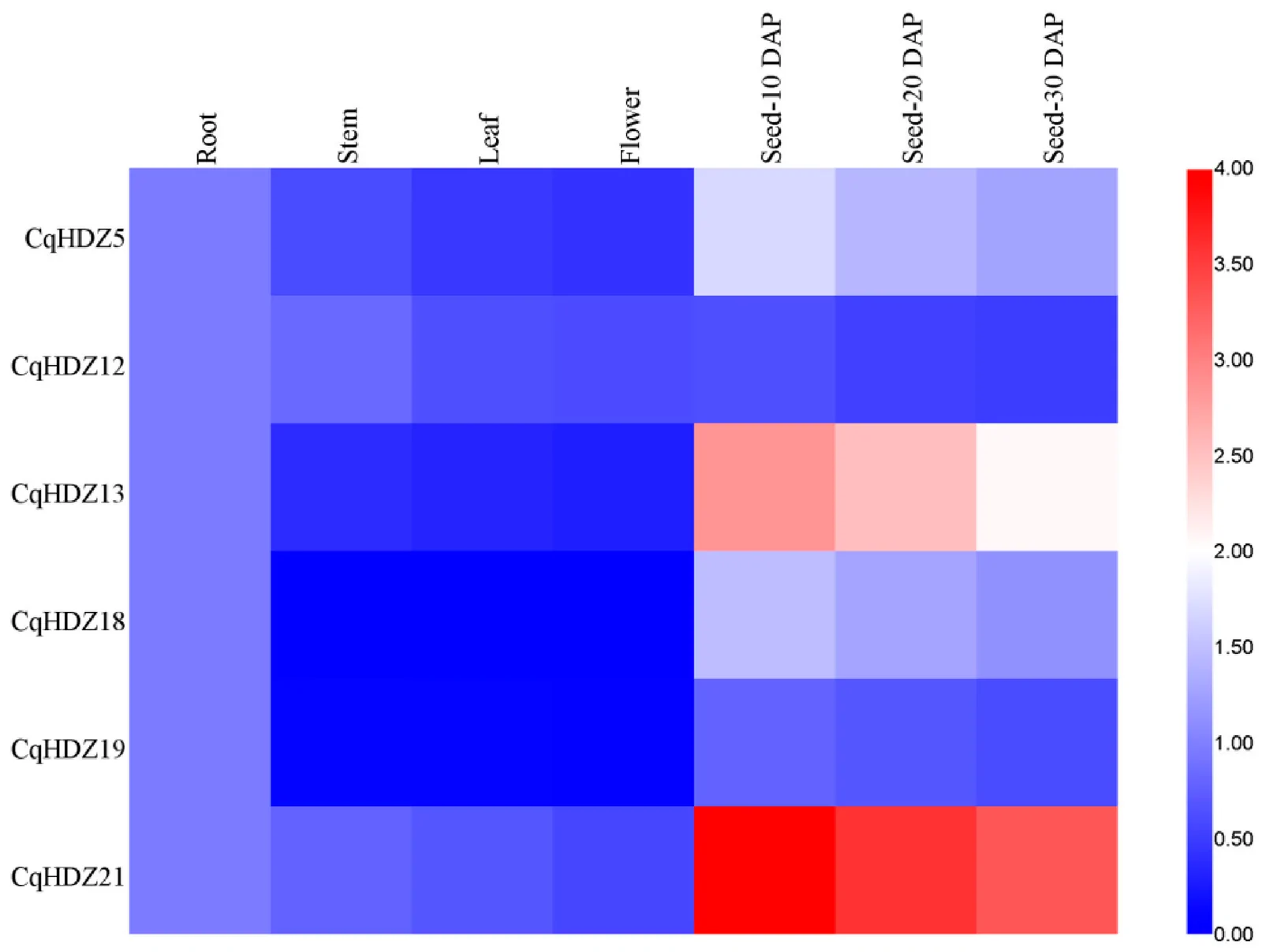
Expression profile of *CqHD-Zip* genes in different tissues and seed development stages. The X-axis represents samples, including root, stem, leaf, flower, seeds 10 days after pollination (seed-10 DAP), seed-20 DAP, and seed-30 DAP; the Y-axis represents *CqHD-Zip* gene names. Red indicates high relative gene expression, whereas blue indicates low relative gene expression.

For more insights into the potential roles of *CqHD-Zip* genes in quinoa growth and development, the relative expression levels of the selected six salt-induced genes were then analyzed in different tissues and seed development stages by qRT-PCR. We found that the *CqHD-Zip* genes exhibited broad tissue expression, suggesting that they might play different regulatory roles. As shown in Figure 7, four genes (*CqHDZ5*, *CqHDZ13*, *CqHDZ18*, and *CqHDZ21*) showed the highest expression levels in seeds of all developmental stages, while the other two genes (*CqHDZ12* and *CqHDZ19*) showed the highest expression levels in roots. Although *CqHDZ18* and *CqHDZ19* had higher relative expression in seeds and roots, they showed extremely low or no expression levels in stems, leaves, and flowers. These expression patterns imply that *CqHD-Zip* genes are potentially involved in seed and root development of quinoa, and functional validation is required to verify their biological functions.

### 3.7. Expression Analysis of CqHD-Zip Genes in Response to Other Abiotic Stresses

**Figure 8:**
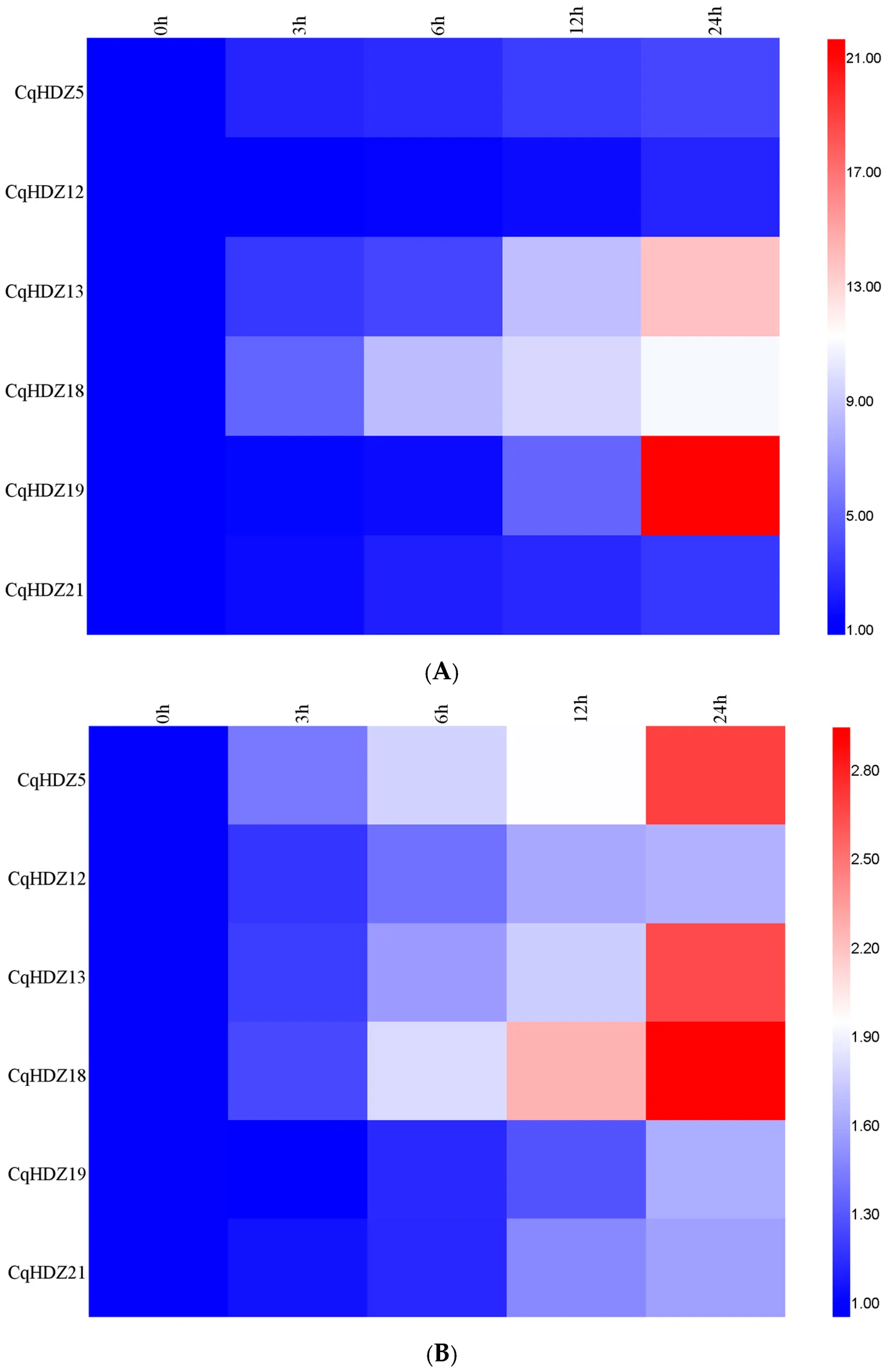
Differential expression of the *CqHD-Zip* genes in response to osmotic stress (**A**) and cold stress (**B**). The relative expression levels were calculated by qRT-PCR (2^−△△CT^). The X-axis represents different time points of stress treatments; the Y-axis represents *CqHD-Zip* gene names. Red indicates high relative gene expression, whereas blue indicates low relative gene expression.

Previous studies have shown that HD-Zip family genes play a critical role in responding to abiotic stresses. To investigate the RNA transcript levels of *HD-Zip* genes in the roots of quinoa seedlings, the expression of the *CqHD-Zip* genes was detected under osmotic and cold conditions using qRT-PCR. After osmotic stress treatment, these six investigated genes were confirmed to show differential accumulation. Interestingly, all the genes were significantly induced, and the expression levels peaked at 24 h, up to 3.98-, 2.71-, 13.93-, 11.14-, 21.79-, and 3.41-fold, respectively (Figure 8A). Similarly, the six genes were up-regulated after cold stress treatment compared with the control (0 h sample), and the expression levels reached their highest at 24 h, showing an ultimate increase to 2.71-, 1.66-, 2.67-, 2.96-, 1.64-, and 1.59-fold, respectively (Figure 8B). Although the fold changes under cold stress are modest, these slight expression shifts could be functionally relevant. The results in this study demonstrated that *CqHDZ5*, *CqHDZ12*, *CqHDZ13*, *CqHDZ18*, *CqHDZ19*, and *CqHDZ21* may play important roles in the response to osmotic and cold stress.

## 4. Discussion

Unfavorable environmental conditions, such as salinity, heat, cold, drought, oxidative stress, and toxic metals, have destructive effects on the growth and development of plant species, which could dramatically reduce crop yield and quality, resulting in significant economic losses [37]. It is estimated that approximately 20% and 22% of the world’s arable land is affected by drought and salinization, reducing crop yields in affected areas by more than 50% [38,39]. To better adapt to adverse circumstances, plants have evolved complicated regulatory pathways to deal with various stimuli, including stress sensing, signal transduction, and inducing the expression of stress-responsive genes, ultimately manifested at the cellular and physiological levels. With the completion of plant genome sequencing and the publication of whole-genome database, more and more stress-responsive genes have been identified, among which identification and analysis of plant gene families is an effective means to mine key functional genes. As key molecular switches, transcription factors regulate physiological and biochemical processes by turning on or inhibiting many genes in response to changes in the external environment to maintain the normal growth and survival of plants [40,41]. Plant-specific HD-Zip transcription factors are considered essential for the regulation of plant development and response against various environmental stresses [6,7,21]. Quinoa is an important pseudocereal crop that can play a strategic role in the development of degraded land in arid areas and saline-alkali land in coastal areas due to its high nutritional value, genetic diversity, and high adaptability to stressful environments [42]. A previous genome-wide survey of the quinoa homeobox superfamily has identified HD-Zip candidates as part of this large transcription factor family [43]. However, a focused, refined inventory and systematic functional characterization of the quinoa HD-Zip family remain limited. In the present study, a comprehensive identification and analysis of *CqHD-Zip* genes from quinoa were conducted. A total of 41 members were identified, which is 7 more than the previous study [43]. There are 30 overlapping genes between the two studies. We newly identified 11 HD-Zip genes, and this difference is attributed to the 11 newly identified genes in our study after removing the inconsistent ones. The phylogenetic relationships, chromosomal distribution, gene structure, conserved motifs, and the expression profiles under various abiotic stresses and in different tissues of this gene family were also analyzed. The results provide valuable information for further study on the function of *CqHD-Zip* genes.

Here, a total of 41 *HD-Zip* genes were identified in the quinoa genome database through bioinformatics methods, named *CqHDZ1* to *CqHDZ41* based on their chromosomal location. The number of *HD-Zip* genes in quinoa is similar in pepper (40 members), more than in rice (31 members), but lower than in Arabidopsis (48 members) and corn (55 members) [15,44,45]. This might be due to sequence duplication events occurring during genomic evolution. The length range of the CqHD-Zip proteins was 182 to 848, the variation range of MW was 20.91 kDa to 92.30 kDa, and the pI range was 4.62 to 9.37. The results of subcellular localization prediction showed that most CqHD-Zip family members were localized in the nucleus, and some were distributed in the cytoplasm, which could act as transcription factors to regulate downstream target genes, consistent with previous studies [12,46]. Furthermore, CqHDZ3, CqHDZ31, and CqHDZ37 were chloroplast-localized transcription factors, indicating their potential functions in plant photosynthesis. Chromosome analysis showed that the 41 *CqHD-Zip* genes were randomly distributed on 16 chromosomes.

In order to explore the evolutionary relationship of HD-Zip proteins, we performed a phylogenetic analysis of HD-Zip homologs from quinoa and Arabidopsis. The CqHD-Zip family can be classified into four subfamilies: HD-Zip I, HD-Zip II, HD-Zip III, and HD-Zip IV, in agreement with the results from previous studies [8,47]. Multiple sequence alignments and phylogenetic trees showed the conservation and high homology of these genes. HD-Zip I subfamily in Arabidopsis has more diverse compositions than the other three subfamilies, as it may be evolutionarily more ancient, which allows more time for gene duplication and rearrangement [48,49]. In quinoa, HD-Zip I is also the most abundant subfamily with the largest number of members, which is in line with previous research. It is reported that HD-Zip III is the most conserved subfamily among various species [50]. Our phylogenetic results also support the high conservation of quinoa HD-Zip III proteins.

The structural characteristics provide additional information for phylogenetic analysis and play a vital role in the evolution of gene families [51,52], while the conserved motifs can reflect their specific biological functions [53]. In this study, the intron/exon structure and conserved motifs were also analyzed; we found that CqHDZs in the same subfamily of the phylogenetic tree are similar in gene structure and motif arrangement, indicating that the functions of *CqHDZs* in different subfamilies change gradually during evolution, thereby helping organisms adapt to external environmental variation. The results further confirmed the accuracy of subgroup classification of the phylogenetic tree. Some motifs appeared more frequently, such as motif 1, motif 2, and motif 3, revealing their importance to the function of CqHD-Zip proteins. The members in CqHD-Zip I and CqHD-Zip II have relatively fewer introns and a different motif composition than in CqHD-Zip III and CqHD-Zip IV subfamilies. Furthermore, CqHD-Zip I proteins have high homology with CqHD-Zip II. Previous studies have noted a potential association between intron number and environmental responsiveness of plant genes [54]. Combined with the intron characteristics observed here, CqHD-Zip I and CqHD-Zip II members may participate in the response to biotic and abiotic stresses.

A vast number of studies found that HD-Zip I proteins played an extremely important role in the response to various abiotic stresses in plants, followed by fewer HD-Zip II subfamily proteins [55,56,57]. Generally, the expression analysis of genes is essential for providing clues for functional prediction [58]. Based on our transcriptomic data, obvious differences existed in the expression of different *CqHDZs* under salt stress. 7 genes were found not to participate in the salt stress response, while the other 34 genes were differentially expressed during salt stress. Among which, the expression levels of some *CqHDZs* changed irregularly at different stress time points; they might be up-regulated at one point and down-regulated at another. In addition, *CqHDZ5*, *CqHDZ12*, *CqHDZ13*, *CqHDZ18*, *CqHDZ19*, and *CqHDZ21* showed continuous upregulation under salt treatment, implying their potential functions as salt stress-responsive regulators. To confirm the reliability of our RNA-Seq data, qRT-PCR was performed on the six genes, and the results were highly consistent. Interestingly, *CqHDZ5*, *CqHDZ13*, *CqHDZ18,* and *CqHDZ19* belonged to the HD-Zip I subfamily, and *CqHDZ12* and *CqHDZ21* belonged to the HD-Zip II subfamily, in accordance with previous results. Then we analyzed the expression patterns of these six genes in different quinoa tissues and under osmotic stress and low-temperature stress. They exhibited tissue- and organ-specific expression profiles, most of which were highly expressed in seeds and roots. The qRT-PCR results showed that all the genes were significantly up-regulated in response to osmotic and cold treatments, and the variation trend of their transcript levels was similar in the two different treatments. This finding indicates the potential function of these genes in osmotic stress and low-temperature stress response in quinoa.

This study characterized the expression patterns of *CqHDZ* genes based on short-read RNA-seq data. It should be noted that quinoa is an allotetraploid species, and many *CqHDZ* genes exist as highly similar homeologous copies. Short sequencing reads derived from conserved regions shared by homeologs tend to map to multiple genomic loci, which limits the ability of short-read data to accurately resolve homeolog-specific expression. Therefore, the expression results presented here cannot completely distinguish transcriptional signals from individual homeologs. To reliably assign transcripts to specific homeologs, verify gene models, and identify alternative splicing isoforms, full-length transcript sequencing [59,60], ideally integrated with short-read quantification, will be required in future investigations.

Collectively, CqHD-Zip is a multifunctional gene family: *CqHDZ5*, *CqHDZ12*, *CqHDZ13*, *CqHDZ18*, *CqHDZ19*, and *CqHDZ21* were highly expressed in quinoa seeds and roots, and might play important roles in the response to abiotic stresses such as salt, osmosis, and cold. Our findings would not only lay a foundation for further understanding the regulatory mechanisms of CqHD-Zip proteins, but also provide a new view for the genetic improvement of resistance in quinoa.

## 5. Conclusions

In this study, a total of 41 HD-Zip transcription factors were identified in the quinoa genome, which were subsequently divided into subfamilies I, II, III, and IV. The physicochemical properties, chromosomal locations, exon-intron structures, conserved motifs, phylogenetic relationships, and expression were comprehensively analyzed. These findings provide a strong foundation for further studies of the role of HD-Zip genes in abiotic stress responses and genetic improvement of quinoa.

## Figures and Tables

**Table 1:** Characteristics of *CqHD-Zip* genes in quinoa.

Gene	Gene ID	Chromosome Location	Exon	ORF	AA	MW (kDa)	pI	Subcellular Localization
*CqHDZ1*	AUR62021994	Chr01:37350019..37356847	10	2205	734	80.53	5.69	Nuclear
*CqHDZ2*	AUR62009262	Chr01:109611250..109617699	17	2508	835	91.90	5.70	Cytoplasmic
*CqHDZ3*	AUR62023427	Chr01:113417905..113419634	3	687	228	25.55	8.71	Chloroplast
*CqHDZ4*	AUR62004302	Chr01:117466602..117471598	8	2334	777	84.78	5.86	Nuclear
*CqHDZ5*	AUR62004541	Chr01:120022607..120023722	2	609	202	23.24	4.83	Nuclear
*CqHDZ6*	AUR62012660	Chr02:2745894..2752727	10	2187	728	79.84	5.69	Nuclear
*CqHDZ7*	AUR62042444	Chr04:9744606..9751218	17	2475	824	90.69	5.86	Cytoplasmic
*CqHDZ8*	AUR62031613	Chr04:39216421..39220923	3	765	254	28.71	6.60	Nuclear
*CqHDZ9*	AUR62004911	Chr05:67114860..67119738	3	876	291	33.55	5.72	Nuclear
*CqHDZ10*	AUR62028812	Chr06:4083901..4091115	3	633	210	23.40	6.65	Nuclear
*CqHDZ11*	AUR62003264	Chr06:10053119..10055330	3	1017	338	38.34	4.97	Nuclear
*CqHDZ12*	AUR62003152	Chr06:11424371..11425602	3	858	285	31.40	8.32	Nuclear
*CqHDZ13*	AUR62026453	Chr06:67574125..67576795	3	969	322	36.52	5.12	Nuclear
*CqHDZ14*	AUR62040157	Chr07:314762..315923	2	570	189	22.07	9.21	Nuclear
*CqHDZ15*	AUR62032758	Chr07:28527556..28541880	5	933	310	35.20	9.37	Nuclear
*CqHDZ16*	AUR62016015	Chr07:77849804..77856632	10	2271	756	83.38	6.00	Nuclear
*CqHDZ17*	AUR62021744	Chr08:8420435..8423555	3	1002	333	37.09	4.65	Nuclear
*CqHDZ18*	AUR62003712	Chr09:5367863..5369745	3	645	214	24.44	8.34	Nuclear
*CqHDZ19*	AUR62022715	Chr10:6071781..6073389	2	759	252	29.18	5.05	Nuclear
*CqHDZ20*	AUR62022751	Chr10:6421904..6427392	8	2289	762	83.22	5.84	Nuclear
*CqHDZ21*	AUR62013727	Chr10:13295738..13297302	3	564	187	21.17	9.01	Nuclear
*CqHDZ22*	AUR62001044	Chr12:13338653..13340678	2	690	229	26.38	5.26	Nuclear
*CqHDZ23*	AUR62007346	Chr13:2025470..2030712	10	2385	794	88.42	5.90	Nuclear
*CqHDZ24*	AUR62007123	Chr13:4727500..4729367	4	1113	370	40.98	9.01	Nuclear
*CqHDZ25*	AUR62007058	Chr13:5608432..5609103	2	573	190	21.89	5.46	Nuclear
*CqHDZ26*	AUR62027204	Chr14:646814..649183	3	1014	337	38.28	5.06	Nuclear
*CqHDZ27*	AUR62007975	Chr14:2231761..2232847	3	795	264	29.07	8.07	Nuclear
*CqHDZ28*	AUR62005746	Chr14:56316232..56317437	2	636	211	23.98	7.80	Nuclear
*CqHDZ29*	AUR62031889	Chr15:16747188..16757208	18	2508	835	91.88	5.96	Cytoplasmic
*CqHDZ30*	AUR62018023	Chr15:21785971..21787998	3	645	214	24.49	8.34	Nuclear
*CqHDZ31*	AUR62024965	Chr15:46953701..46961292	19	2463	820	89.59	5.81	Chloroplast
*CqHDZ32*	AUR62008353	Chr16:3535903..3538215	3	1023	340	37.96	4.62	Nuclear
*CqHDZ33*	AUR62019921	Chr16:72846053..72846719	2	549	182	20.91	5.45	Nuclear
*CqHDZ34*	AUR62019992	Chr16:73618172..73620059	4	1110	369	40.66	9.08	Nuclear
*CqHDZ35*	AUR62018653	Chr16:76516708..76521772	12	2220	739	82.05	5.99	Nuclear
*CqHDZ36*	AUR62037903	Chr17:2416074..2418417	3	675	224	26.09	8.92	Nuclear
*CqHDZ37*	AUR62042187	Chr18:18015511..18022900	18	2547	848	92.30	5.99	Chloroplast
*CqHDZ38*	AUR62022184	Chr18:20987134..20997210	18	2508	835	91.88	5.97	Cytoplasmic
*CqHDZ39*	AUR62021220	Chr00:35823613..35830897	8	2304	767	84.49	5.63	Nuclear
*CqHDZ40*	AUR62044008	Chr00:79088942..79095440	19	2205	734	81.06	6.24	Cytoplasmic
*CqHDZ41*	AUR62044145	Chr00:102819093..102821119	3	645	214	24.49	8.34	Nuclear

## Data Availability

The original contributions presented in this study are included in the article. Further inquiries can be directed to the corresponding authors.

## Supplementary Materials

The following supporting information can be downloaded at: https://www.mdpi.com/article/10.3390/biology15212012/s1, Table S1: Conserved domains of CqHD-Zips in quinoa; Table S2: Multiple sequence alignment of 41 CqHD-Zip proteins; Table S3: Primer sequences for qRT-PCR.
